# Prognostic role of the beta-2 adrenergic receptor in clear cell renal cell carcinoma

**DOI:** 10.1080/19768354.2019.1658638

**Published:** 2019-08-25

**Authors:** Mihyang Ha, Dong Woo Kim, Jayoung Kim, Chae Mi Hong, Su Min Park, In Ae Woo, Min Yong Kim, Hyunjun Koo, Jin Namkoong, Jaehyun Kim, Myoung-Eun Han, Parkyong Song, Jin Hur, Chi-Dug Kang, Yun Hak Kim, Dongjun Lee, Sae-Ock Oh

**Affiliations:** a Department of Anatomy, Pusan National University School of Medicine, Yangsan, Republic of Korea; b Department of Premedicine, Pusan National University School of Medicine, Yangsan, Republic of Korea; c Department of Convergence Medicine, Pusan National University School of Medicine, Yangsan, Republic of Korea; d Department of Anatomy, Department of Biomedical Informatics, and Biomedical Research Institute, Pusan National University School of Medicine, Yangsan, Republic of Korea

**Keywords:** ADRB2, ICGC, TCGA, ccRCC

## Abstract

The beta-2 adrenergic receptor (ADRB2) regulates the proliferation, apoptosis, angiogenesis, migration, and metastasis of cancer cells. However, its function in the progression of clear cell renal cell carcinoma (ccRCC) is unknown. Here, we report that ADRB2 can be a novel prognostic factor for patients with ccRCC. The differential expression of ADRB2 in low-stage (stages I and II), high-stage (stages III and IV), low-grade (grades I and II), and high-grade (grades III and IV) ccRCC was identified in cohorts of patients from The Cancer Genome Atlas and the International Cancer Genome Consortium. We evaluated *ADRB2* expression as a prognostic factor using the Kaplan-Meier survival curve, multivariate analysis, time-dependent area under the curve (AUC) of Uno’s C-index, and AUC of the receiver operating characteristics (ROC) at five years. Kaplan-Meier analysis revealed that reduced *ADRB2* expression is associated with poor prognosis in ccRCC patients. Analysis of C-indices and AUC-ROC further confirmed this result. Moreover, multivariate analysis confirmed the prognostic significance of *ADRB2* expression. Collectively, these findings suggest that ADRB2 is a potential prognostic factor for ccRCC.

## Introduction

Clear cell renal cell carcinoma (ccRCC) is the most prevalent subtype of kidney cancer and approximately 30% of kidney cancer patients present with metastasis (Nickerson et al. 2008). In addition, approximately 30% of ccRCC patients have been diagnosed with advanced disease (Karakiewicz et al. 2007). Current therapeutic treatments against renal cancer are not sufficiently effective; therefore, novel biomarkers for ccRCC that could provide prognostic information for clinical use are required. Moreover, prognostic biomarkers for ccRCC have been investigated in cohorts of patients from The Cancer Genome Atlas (TCGA) (Cerami et al. 2012; Cancer Genome Atlas Research et al. 2013) and the International Cancer Genome Consortium (ICGC) (International Cancer Genome et al. 2010).

Beta-adrenergic receptors (βARs) are G protein-coupled receptors that regulate various cellular processes, including proliferation, invasion, and activation of the immune response (Barron et al. 2012). βARs are expressed on tumor cells and stromal cells in the tumor microenvironment (Sloan et al. 2010; Powe et al. 2011), and stress-induced βAR activation recruits immune cells to primary tumors (Sloan et al. 2010). Moreover, the activation of βARs can reduce tumor cell proliferation and primary tumor growth *in vivo* (Carie and Sebti 2007). The beta-2 adrenergic receptor (ADRB2) is the most abundant receptor for sympathetic signaling in prostate luminal cells (Braadland et al. 2014). ADRB2 expression was decreased during prostate cancer metastasis (Yu et al. 2007). However, the clinical and prognostic significance of ADRB2 in ccRCC remain unknown. In this study, we present the first data on *ADRB2* expression in cohorts of patients with well-defined primary ccRCC from TGCA and ICGC and ADRB2 can be an important prognostic factor of ccRCC.

## Materials and methods

### Patient data acquisition and statistical analysis

The clinical and genomic data were acquired from TCGA and the ICGC data portal (dcc.icgc.org) on March 2018. Samples with insufficient survival data were excluded, as previously described (Han et al. 2018; Ha et al. 2019).

Overall survival (OS) prediction and associated statistical analyses were performed using R software version 3.5.0 (The R Foundation for Statistical Computing). The following statistical methods were used for analyses: (1) Uno’s C-index, (2) area under the curve (AUC) values at five years, and (3) *p*-value from log-rank test to evaluate the accuracy of the discrimination, as described previously using ‘survival’ and ‘survAUC’ R packages (Cho et al. 2018; Han et al. 2018). The C-index is a well-known parameter of the fit of a survival model, in continuous time, within a clinical study (Uno et al. 2011; Kim, Jeong, Pak, Goh, et al. 2017; Kim, Jeong, Pak, Han, et al. 2017). In the Kaplan-Meier analyses, we determined the optimal cut-off value (TCGA: 31.5365 and ICGC: 0.732) that had the maximal Uno’s C-index by five-fold cross-validation (Table 1) (Cho et al. 2018; Han et al. 2018; Ha et al. 2019). Univariate and multivariate Cox regression analysis was performed to assess the effect of *ADRB2* expression as a categorical value on prognosis, along with other clinical variables (Table 2).

**Table 1.** C-index and area under the curve (AUC) values for *ADRB2* in the specified categories in TCGA or ICGC cohorts.

Category	C-index		AUC value at 5 years	
	TCGA	ICGC	TCGA	ICGC
All patients	0.605	0.677	0.588	0.642
Stages I & II	0.543	0.442	0.531	0.521
Stages III & IV	0.577	0.758	0.572	0.777
Grades I & II	0.521	–	0.502	–
Grades III & IV	0.600	–	0.602	–

TCGA: The Cancer Genome Atlas; ICGC: International Cancer Genome Consortium.

**Table 2.** Univariate and multivariate analysis of overall survival in each cohort (*P < 0.05, ** *P* < 0.01, *** *P* < 0.001).

	Univariate analysis				Multivariate analysis			
Parameters	*P*	HR	95 Cl		*P*	HR	95 Cl	
TGCA								
ADRB2	<0.001***	0.458	0.324	0.638	<0.001***	0.532	0.375	0.755
Age	<0.001***	1.033	1.018	1.047	<0.001***	1.030	1.015	1.046
Stage (I, II vs. III, IV)	<0.001***	3.478	2.474	4.888	<0.001***	2.730	1.903	3.917
Gender (Female vs. Male)	0.333	0.850	0.612	1.181	0.569	0.904	0.640	1.278
Grade (I, II vs. III, IV)	<0.001***	2.247	1.572	3.212	0.040*	1.486	1.019	2.168
ICGC								
ADRB2	<0.001***	0.299	0.146	0.614	0.003**	0.302	0.137	0.666
Age	0.109	1.031	0.993	1.071	0.157	1.028	0.990	1.067
Stage (I, II vs. III, IV)	<0.001***	4.796	2.264	10.16	<0.001***	4.282	1.978	9.269
Gender (Female vs. Male)	0.863	1.066	0.517	2.194	0.758	1.130	0.518	2.466

TCGA: The Cancer Genome Atlas; ICGC: International Cancer Genome Consortium; ADRB2: Beta-2 adrenergic receptor.

## Results

### Downregulation of ADRB2 in high-stage and high-grade patients with ccRCC

**Figure 1.**
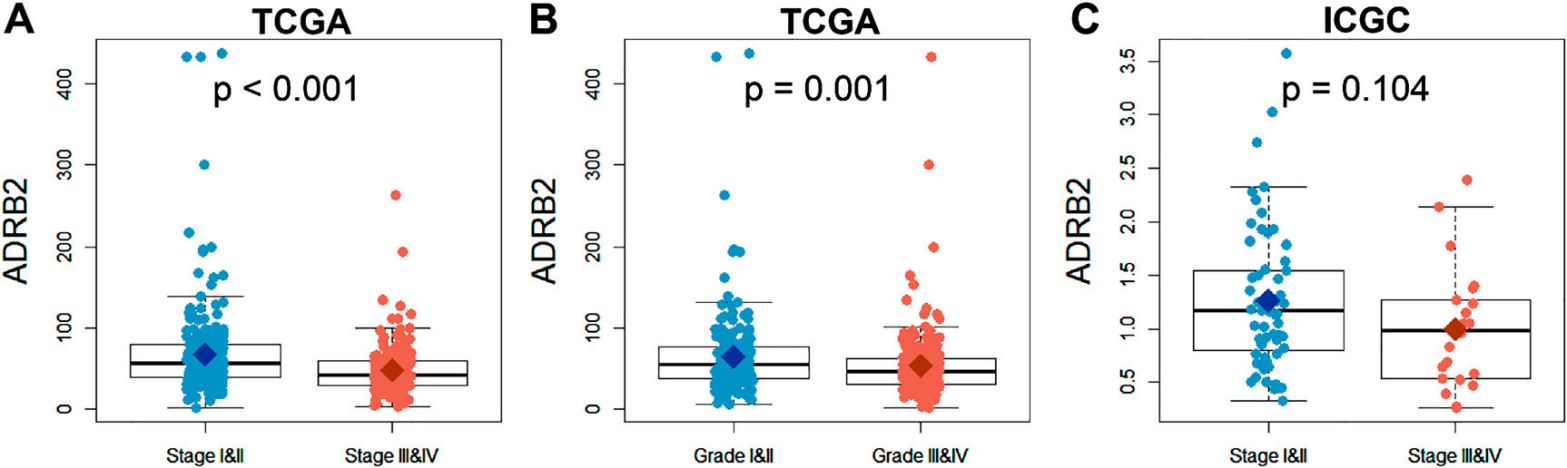
Comparison of *ADRB2* expression among low-stage (stages I and II), high-stage (stages III and IV), low-grade (grades I and II), and high-grade (grades III and IV) patients in TCGA and ICGC cohorts. (A and B) *ADRB2* expression in patients with ccRCC in TCGA cohort. (C) *ADRB2* expression in patients with ccRCC in ICGC cohort.

In total, 446 patients from TCGA and 91 from the ICGC were included in this study. Patient information is summarized in Table 3. *ADRB2* expression was compared between low-stage (stages I and II) and high-stage (stages III and IV) cohorts of patients with ccRCC from TCGA and ICGC, and between low-grade (grades I and II) and high-grade (grades III and IV) cohorts of patients with ccRCC from TCGA, respectively. *ADRB2* expression in the low-stage and low-grade ccRCC cohorts was considerably higher than that in the high-stage and high-grade cohorts (Figure 1).

**Table 3.** Patient characteristics in TCGA or ICGC cohorts.

		TCGA (%)	ICGC (%)
Stage	I	216 (48.4)	48 (52.7)
	II	46 (10.3)	12 (13.2)
	III	111 (24.9)	13 (14.3)
	IV	71 (15.9)	9 (9.9)
	NA	2 (0.4)	9 (9.9)
Grade	I	9 (2.0)	–
	II	189 (42.4)	–
	III	175 (39.2)	–
	IV	68 (15.2)	–
	NA	5 (1.1)	–
Sex	Male	290 (65.0)	52 (57.1)
	Female	156 (35.0)	39 (42.9)
Age (mean ± standard deviation)		60.62 ± 12.80	60.47 ± 10.03
Total number of patients		446	91

TCGA: The Cancer Genome Atlas; ICGC: International Cancer Genome Consortium.

### The prognostic value of ADRB2 expression in ccRCC patients

**Figure 2.**
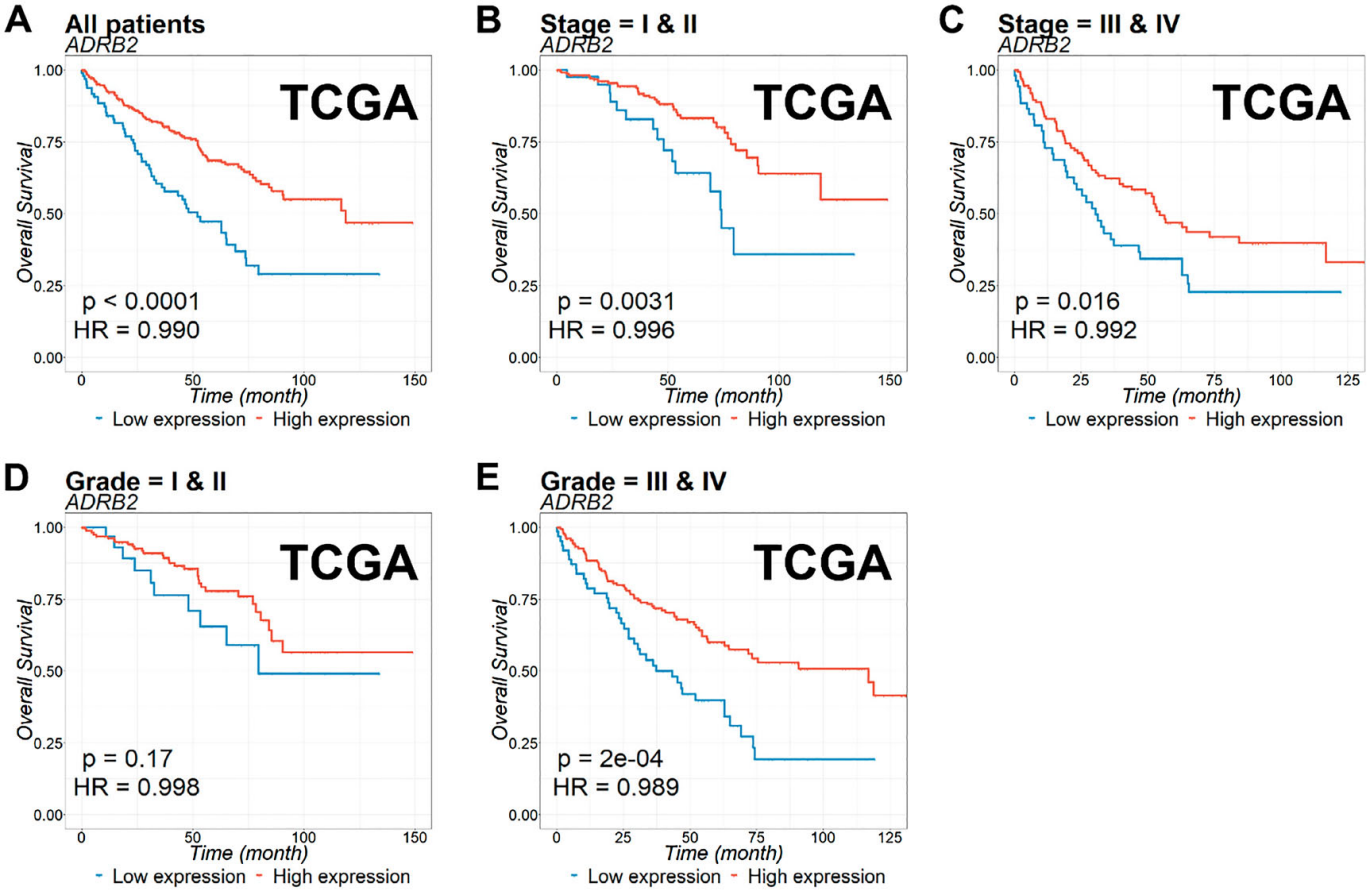
Kaplan-Meier estimation of overall survival (OS) for patients with ccRCC according to *ADRB2* expression. OS of (A) all patients, or patients with (B) stages I and II, (C) stages III and IV, (D) grades I and II, and (E) grades III and IV ccRCC in TCGA cohort was examined based on *ADRB2* expression. The *p*-value was calculated using the log-rank test and is described on the bottom left.

**Figure 3.**
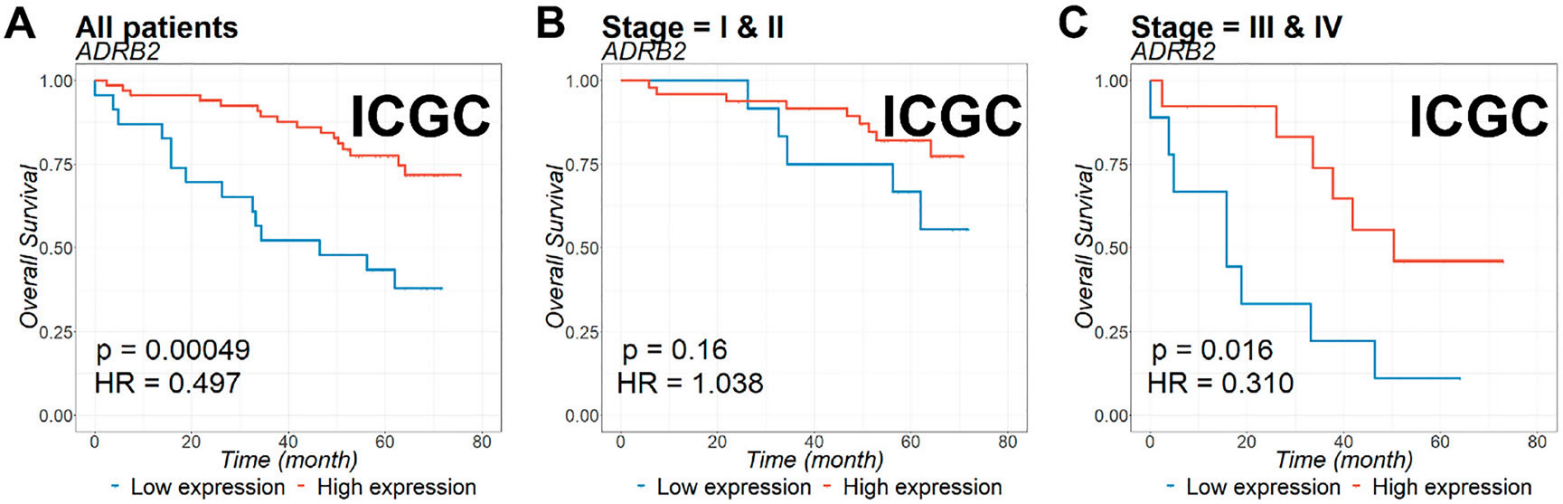
Kaplan-Meier estimation of overall survival (OS) for patients with ccRCC according to *ADRB2* expression. OS of (A) all patients, or patients with (B) stages I and II, and (C) stages III and IV ccRCC in ICGC cohort was examined based on *ADRB2* expression. The *p*-value was calculated using the log-rank test and is described on the bottom left.

To evaluate the prognostic value of *ADRB2* in ccRCC, we analyzed Kaplan-Meier curves for *ADRB2* gene expression and OS in TCGA (Figure 2) and ICGC (Figure 3) cohorts. Low expression of *ADRB2* correlated with significantly shorter OS than did the high expression of *ADRB2* in TCGA (Figure 2) and ICGC cohorts (Figure 3). The prognostic value was further confirmed using multivariate analysis (*P *<* *0.001 and *P* = 0.003 for TCGA and ICGC, respectively, in Table 2).

**Figure 4.**
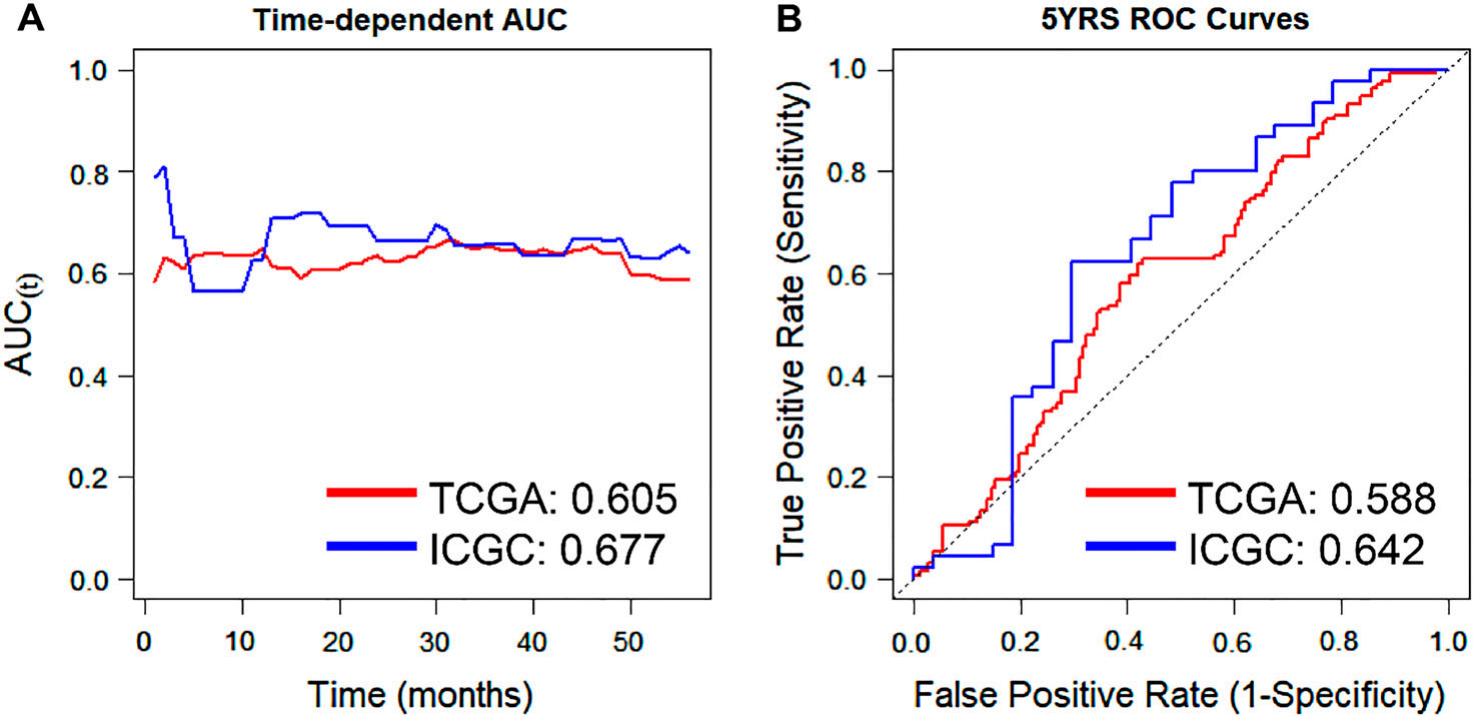
Time-dependent area under the curve (AUC) and receiver operating characteristics (ROC) curves at five years based on *ADRB2* expression in TCGA and ICGC cohorts. (A) Time-dependent AUC curve and (B) ROC curve at five years in TCGA and ICGC cohorts based on *ADRB2* expression. C-index values are described on the bottom right of (A). AUC values at five years are described on the bottom right of (B).

To assess the validity of *ADRB2* expression as a prognostic factor for ccRCC, we assessed Uno’s C-index from time-dependent AUC analysis and AUC at five years for receiver operating characteristics (ROCs) in TCGA and ICGC cohorts (Figure 4). *ADRB2* exhibited high C-index values in the two independent cohorts (TCGA: 0.605 and ICGC: 0.677; Figure 4A and Table 1). The five-year ROC graphs revealed high AUC values in TCGA and ICGC cohorts (TCGA: 0.588 and ICGC: 0.642; Figure 4B and Table 1).

## Discussion

In this study, we identified *ADRB2* expression as a prognostic factor for ccRCC, and demonstrated that reduced expression of *ADRB2* is associated with poor patient prognosis. The current therapeutic treatment of ccRCC has a low rate of success (Subramanian and Haas 2018). Although there are many treatment options for ccRCC, surgical intervention is the most effective method to treat clinically localized ccRCC. Despite the availability of advanced surgical and medical techniques, ccRCC recurrence and metastasis rates remain high because of micro-environmental changes (Subramanian and Haas 2018; Wang et al. 2018). Transcriptome-based prognostic factors have been identified in many cancers, some of which have shown a sufficiently satisfactory outcome based on clinical guidelines (van ‘t Veer et al. 2002; Paik et al. 2004; Nault et al. 2013; Kim, Jeong, Pak, Goh, et al. 2017; Kim, Jeong, Pak, Han, et al. 2017). Therefore, novel molecular markers can be used in combination with current staging systems.

In summary, the main purpose of our study was to expand the foundation of precision medicine by analyzing big genome data. Our results showed that *ADRB2* expression is inversely correlated with patient prognosis in both examined cohorts. Although there are limitations in transcriptome-based studies of *ADRB2*, we believe that there is sufficient evidence to suggest that *ADRB2* can act as a prognostic biomarker in ccRCC.
